# Immunogenicity of a Multi-Epitope DNA Vaccine Encoding Epitopes from Cu–Zn Superoxide Dismutase and Open Reading Frames of *Brucella abortus* in Mice

**DOI:** 10.3389/fimmu.2017.00125

**Published:** 2017-02-09

**Authors:** Emilia Escalona, Darwin Sáez, Angel Oñate

**Affiliations:** ^1^Laboratory of Molecular Immunology, Department of Microbiology, Faculty of Biological Sciences, Universidad de Concepción, Concepción, Chile

**Keywords:** brucellosis, *Brucella abortus*, multi-epitope DNA vaccine, genomic island 3, Cu–Zn SOD

## Abstract

Brucellosis is a bacterial zoonotic disease affecting several mammalian species that is transmitted to humans by direct or indirect contact with infected animals or their products. In cattle, brucellosis is almost invariably caused by *Brucella abortus*. Live, attenuated *Brucella* vaccines are commonly used to prevent illness in cattle, but can cause abortions in pregnant animals. It is, therefore, desirable to design an effective and safer vaccine against *Brucella*. We have used specific *Brucella* antigens that induce immunity and protection against *B. abortus*. A novel recombinant multi-epitope DNA vaccine specific for brucellosis was developed. To design the vaccine construct, we employed bioinformatics tools to predict epitopes present in Cu–Zn superoxide dismutase and in the open reading frames of the genomic island-3 (BAB1_0260, BAB1_0270, BAB1_0273, and BAB1_0278) of *Brucella*. We successfully designed a multi-epitope DNA plasmid vaccine chimera that encodes and expresses 21 epitopes. This DNA vaccine induced a specific humoral and cellular immune response in BALB/c mice. It induced a typical T-helper 1 response, eliciting production of immunoglobulin G2a and IFN-γ particularly associated with the Th1 cell subset of CD4^+^ T cells. The production of IL-4, an indicator of Th2 activation, was not detected in splenocytes. Therefore, it is reasonable to suggest that the vaccine induced a predominantly Th1 response. The vaccine induced a statistically significant level of protection in BALB/c mice when challenged with *B. abortus* 2308. This is the first use of an *in silico* strategy to a design a multi-epitope DNA vaccine against *B. abortus*.

## Introduction

Brucellosis, caused by facultative Gram-negative intracellular coccobacilli grouped in the genus *Brucella* (1), is a zoonotic disease with a high incidence and prevalence worldwide. Brucellosis affects mammals, and it can considerably undermine the health and productivity of domestic livestock. The most frequent clinical symptom in livestock after *Brucella* infection is abortion (2). In humans, the disease has a wide spectrum of clinical manifestations. They range from simple fever to major complications in which function in the nervous, digestive, genital-urinary, cardiovascular, and muscular systems is compromised, sometimes leading to death (3). Brucellosis can impose a significant economic burden on animal production (reduction in milk production, abortions, delayed in conception). In cattle, it has been estimated that more than 300,000 animals, out of the 1.4 billion in the world, are infected (4). Brucellosis is one of the most common zoonotic diseases in humans, with more than 500,000 cases reported annually. However, depending upon the system of controls and the socioeconomic conditions, official reports only account for a fraction of the true incidence of this disease, and different countries have reported from 0.09 to 1603 cases per million inhabitants (5).

Infection by *Brucella* spp. is usually associated to an acute inflammatory reaction, the principal mechanism against local proliferation of *Brucella* organisms. Infection initially prompts an innate immune response that reduces the number of bacteria (6). The innate response activates immunity mediated by cells, in which CD4+ and CD8+ T lymphocytes, macrophages (MΦ), dendritic cells (DCs), and pro-inflammatory cytokines, such as interferon-gamma (IFN-γ) and tumor necrosis factor-alpha (TNF-α), participate to confer protection (7). Although the host raises strong immune response, *Brucella abortus* has the capacity to survive inside MΦ and DCs, expressing a number of virulence factors that allow it to reach its replicative niche and to avoid immune-mediated destruction (8). It has been shown that antigen O protects *Brucella* from intracellular death mechanisms, while lipid A is involved in evasion of the innate immune system during the first stages of infection (9). Some additional virulence factors have been reported to be involved in intracellular replication and immune evasion. These include VirB type IV secretion (10), BTP1, a seven-helix transmembrane protein that prevents maturation of DCs (11), a BvrR/BvrS regulatory system of two components (12), and the RNA chaperone protein Hfq (13). *B. abortus* contains a Cu–Zn superoxide dismutase (SOD1), an homodimeric metalloenzyme (14). SOD1 catalyzes the dismutation of the superoxide anion ${\text{O}}_{2}^{-}$ to O_2_ and H_2_O_2_, detoxifying superoxide radicals generated during the host antimicrobial immune response (15). It has been observed that a DNA vaccine with the gene sequence for this protein (*sodC*) is highly immunogenic (16, 17).

One characteristic of *Brucella* is a limited genetic diversity. This is manifested by the small number of differences responsible for host preferences and by virulence restrictions (18). The *Brucella* genome incorporates transfer-acquired mobile elements referred to as genomic islands (GIs). Nine GIs have been identified in *Brucella* (19). GI-3 is present in *B. abortus, Brucella melitenses*, and *Brucella ovis*. This GI contains 25 genes, many of which have unknown function, and several pseudogenes (20). GI-3 in *B. abortus* 2308 includes open reading frame (ORF) BAB1_0260, BAB1_0270, BAB1_0273, BAB1_0278, and BAB1_0278a. Our group has reported that BAB1_0270, described as a zinc-dependent metallopeptidase protein, and BAB1_0278, which has homology with the GcrA superfamily, are involved in *Brucella* virulence. Their deletion affects the capacity of *Brucella* to invade phagocytic cells and to survive within them (21, 22). Furthermore, DNA vaccine encoding BAB1_0270 (23), BAB1_0278 (24), and BAB1_0278a, a hypothetical ABC-type transporter (25), were able to induce an immune response and protection against *B. abortus* 2308 infection. Immunization with the recombinant flagellar protein (BAB1_0260) also induced protection (26). Based on bioinformatics analysis BAB1_0273, a possible DNA-binding protein, is a putative antigen.

Despite the existence of effective commercial vaccines against brucellosis and a diagnostic test, it has not been possible to eradicate the disease. The main vaccines currently used for cattle are based on live bacteria attenuated to decrease their pathogenicity. Cattle are often vaccinated with *B. abortus* S19 or RB51, which, although providing good protection, may induce abortion if administered to gravid females (27), and are potentially infectious to humans (28). Recent advances in genomics, proteomics, recombinant DNA techniques, and vaccinology have made possible the development of safer vaccines, which overcome the drawbacks associated with live-attenuated vaccines. For example, DNA vaccines offer the possibility of inducing both humoral and cellular immune responses, and potentially can prolong the expression of an antigen (29). The use of epitopes in the design of this type of vaccine is a new alternative in the development of multi-epitope DNA vaccines (30–32). In this strategy, an informed selection of antigenic determinants that correlate with immunogenicity was used.

In this study, we have predicted antigenic determinants using bioinformatics tools from any ORF codified in GI-3 from *B. abortus* and designed a multi-epitope chimeric DNA vaccine. Humoral, cell-mediated, and protective immunity induced by this multi-epitope DNA vaccine was examined in BALB/c mice.

## Materials and Methods

### Animals

Seven- to eight-week-old female isogenic BALB/c mice (obtained from the Instituto de Salud Pública, Santiago, Chile) were randomly allocated to three groups. Mice were kept in conventional animal facilities and received water and food *ad libitum*. All animals were handled in accordance with the regulations of the Bioethics Committee of the Faculty of Biological Sciences, Universidad de Concepción regulations. The Bioethics and Safety committee of the Faculty of Biological Sciences of the Universidad de Concepción approved this study. All efforts were made to minimize animal suffering.

### Bacterial Strains

*Escherichia coli* strain BL21 (DE3) pLys (Novagen, Madison, WI, USA) was used as the host strain for expression of recombinant multi-epitope protein and *E. coli* DH5α (Invitrogen, San Diego, CA, USA) was used for obtaining plasmids. Both strains were grown at 37°C in LB broth. The virulent *B. abortus* 2308 and the attenuated strain RB51 were obtained from our culture collection. Bacterial cells were grown under aerobic conditions in Trypticase soy broth (Difco Laboratories, Detriot, MI, USA) for 72 h at 37°C.

### Epitope Prediction

The selected protein sequences (Table 1) were obtained from the NCBI Database. To find promising epitopes, we used the Immune Epitope Database (33). This database contains epitope information from 99% of all papers published about immune epitopes (33). We used the T Cell Epitope Prediction Tools to find peptides binding to MHC class I and class II molecules. Epitope prediction was performed for H2-D^d^, H2-K^d^, H2-L^d^ alleles and H2-IE^d^, H2-IA^d^ alleles, and MHC-I and MHC-II haplotypes in BALB/c mice. We used a consensus method consisting of a combination of the Stabilized Matrix Method (34), Artificial Neural Network (35), and Scoring Matrices derived from Combinatorial Peptide Libraries (36). We set a threshold <20 of percentile rank and selected all peptides lower than this.

**Table 1:** ***Brucella abortus* proteins used to design the multi-epitope DNA vaccine**.

Protein name	Locus tag	Position	Genebank ID	Length (aa)
Copper/Zinc superoxide dismutase	BAB2_0535	AM040265.1:534069–534590	CAJ12701.1	173
Flagellar protein FlgJ	BAB1_0260	AM040264.1:263739–265859	CAJ10216.1	706
Zinc-dependent metallopeptidase	BAB1_0270	AM040264.1:270965–271513	CAJ10226.1	182
Hypothetical DNA-binding protein	BAB1_0273	AM040264.1:272920–273165	CAJ10229.1	81
Hypothetical GcrA protein	BAB1_0278	AM040264.1:275602–275838	CAJ10234.1	78
Hypothetical ABC-type transporter	BAB1_0278a	AM040264.1:275322–275654	EEP63779.1	110

### Construction of Recombinant Plasmids

**Figure 1:**
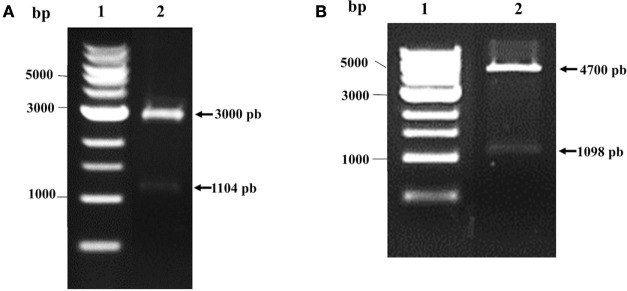
**Verification of pV-MEB DNA vaccine and pQE80L-MEB plasmids**. **(A)** Restriction analysis of pV-MEB plasmid digested with *Bam*HI and *Pst*I (line 1) **(B)** Restriction analysis of pQE80L-MEB plasmid digested with *Bam*HI and *Pst*I (line 1). LM, DNA size marker (1 kb DNA ladder).

pVAX1 vector (Thermo Fisher Scientific Inc., MA, USA), designed for use in the development of DNA vaccines, and pQE80L bacterial laclq vector (Qiagen), for expressing N-terminally 6xHis-tagged proteins, were used to induce the expression of recombinant protein. The multi-epitope genes were chemically synthesized by GenScript, Inc. (Piscataway, NJ, USA), with codon optimization for mouse and *E. coli*. The Kozak or Shine-Dalgarno sequence was included in the respective genes. The genes were inserted into pUC57 to generate two expression vectors: pUC57-MEBe (expressing to the recombinant protein) and pUC57-MEBm (used to construct the DNA vaccine). Both constructs were digested with *Bam*HI-*Pst*I and subcloned into *Bam*HI-*Pst*I-digested pVAX1 or *Bam*HI-*Pst*I-digested pQE80L. We obtained pV-MEB (multi-epitope DNA vaccine for *Brucella*) and pQE80L-MEB plasmids and confirmed them by restriction digestion analysis (Figure 1). We observed 1140 base pairs (bp) corresponding to MEBm fragment (A) and 1098 bp corresponding to MEBe fragment (B), respectively.

### Immunization

BALB/c mice were randomly divided into three groups consisting of 10 mice per group. Group 1 was injected with 100 µg of the pV-MEB vaccine in 100 µl of phosphate buffer saline (PBS), divided into two injections of 50 µl, in each posterior tibialis muscle. As negative controls, groups of mice received either 100 µg pVAX1 in 100 µl of PBS or 100 µl of PBS, injected as described above for the experimental group (23). All groups were immunized three times at 15-day intervals.

### Purification of Recombinant Multi-Epitope Protein

To obtain the multi-epitope recombinant protein, *E. coli* BL-21 were chemically transformed with pQE80L-MEBe, and we standardized the protocols to carry out the purification of the rMEB protein. Transformed bacteria were grown in LB broth at 37°C to mid-log phase [optical density at 600 nm (OD_600_), 0.6–0.8]. To induce the expression of recombinant protein, bacteria were cultured with 0.5 mM Isopropyl β-d-1-thiogalactopyranoside (IPTG) for 4 h at 27°C to induce the expression of recombinant protein. Thereafter, the transformed bacterial cells were collected by centrifugation and then disrupted by sonication in Tris–HCl buffer plus 0.2 mM of phenylmethylsulfonyl fluoride (PMSF). This preparation was centrifuged at 12,000 *g* for 20 min, the soluble fraction was saved and the insoluble fraction was denatured with Denaturing Binding Buffer (0.2 mM PMSF; 20 mM Tris–HCl pH 8; 0.5 M NaCl; 6 M Urea; 10 mM Imidazole). The his-tagged rMEB protein was purified by Ni^2+^-chelated affinity chromatography with HisTrap FF crude columns (GE Healthcare Life Sciences), according to the manufacturer’s instructions. The elution was performed with 100 mM of Imidazole. The eluate was concentrated and desalinated using a filter with a molecular weight exclusion of 10 kDa (Amicon ultra 100 K, Millipore, MA, USA). Protein concentration was determined by the Pierce™ BCA Protein Assay kit (Thermo Fisher Scientific Inc.). Recombinant multi-epitope proteins were stored at −20°C for later use as antigens in a indirect enzyme-linked immunosorbent assay (ELISA) and lymphocyte proliferation assays.

### Humoral Immune Response

The antibody isotype IgG, IgG1, and immunoglobulin G2a (IgG2a) titers were measured from peripheral blood using an ELISA. Serum was collected 2 days before each immunization and 15 days after the last immunization. Ninety-six-well polystyrene microtiter plates (Thermo Fisher Scientific Inc., MA, USA) were coated with 1 µg/ml of rMEB or 10 µg/ml of crude *Brucella* protein (CBP) (37), diluted in 0.05 M carbonate–bicarbonate buffer (pH 9.6). After overnight incubation at 4°C, the plates were blocked with 0.8% gelatin in Tris-buffered saline for 1 h at 37°C. Serial twofold dilutions of sera containing primary antibodies from test and control animals were added and incubated for 3 h at room temperature. Isotype-specific horseradish peroxidase-conjugated anti-mouse IgG (US Biological, Life Sciences) was added at 1:1000 dilution. After 30 min of incubation at room temperature, 200 µl of substrate solution (Sigma-Aldrich, Inc.) was added to each well. Results were read using a VictorX3 Multilabel Plate Reader (PerkinElmer, USA) at 450 nm. All assays were done in triplicate.

### Culture of Splenocytes and Lymphocyte Proliferation

Four weeks after the last immunization, mice were euthanized and their spleens removed under aseptic conditions. The splenocytes were cultured, at a concentration of 4 × 10^6^ viable cells/ml (100 µl per well), at 37°C under 5% CO_2_ in a 96-well flat-bottom plate (Nunc, Denmark), previously sensitized with 2 µg/ml or 10 µg/ml of recombinant proteins (rMEB), or 2 µg/ml or 10 µg/ml CBP. Splenocytes were cultured in RPMI 1640 medium (Thermo Fisher Scientific, MA, USA) supplemented with 10% heat-inactivated fetal calf serum (GIBCO BRL), penicillin–streptomycin (50 UI of penicillin; 50 µg/ml streptomycin), and amphotericin B (0.25 µg/ml). After 72 h, cells were pulsed for 8 h with 0.4 μCi thymidine (50 μCi/mmol; Amersham, UK) per well and the radioactivity incorporated in the DNA measured using a scintillation counter. Concanavalin A (ConA) (Sigma Aldrich, MO, USA), at a concentration of 10 µg/ml was used as proliferative positive control and 10 µg/ml albumin protein or 10 µg/ml total *E. coli* protein (CEP) were used as proliferative negative control. Cell proliferation data were expressed as the stimulation index of triplicate cultures from a cell pool from each group. These were obtained by dividing the amount of ^3^H-Thymidine incorporated (c.p.m.) in antigen-stimulated cell cultured by the c.p.m. obtained from cells cultured without antigen (38).

### Cytokine ELISAs

The levels of IFN-γ and IL-4 secreted were measured by antigen-capture ELISA. Briefly, spleens were aseptically removed from experimental and control mice, disaggregated to single cells, re-suspended in Red Blood Cell buffer (Promega, Madison, WI, USA) to eliminate erythrocytes, and washed three times using incomplete RPMI 1640 (Thermo Fisher Scientific, MA). Cells were adjusted to a concentration of 4 × 10^6^ viable cells per ml in RPMI 1640 supplemented with 10% fetal calf serum (Thermo Fisher Scientific, MA, USA) and antibiotic/antimycotic solution (100 UI penicillin, 100 µg/ml streptomycin, and 0.25 µg/ml amphotericin B). Spleen cell suspensions were cultured in 24-well plates (Nunc, Denmark) and stimulated with the recombinant multi-epitope *B. abortus* protein (rMEB) at 2 or 10 µg/ml CBPs or medium alone. They were incubated for 48 h at 37°C under 5% CO_2_ to induce, *in vitro*, the expression of cytokines. After centrifugation at 400 × *g* for 10 min, supernatants were collected and cytokines quantified by ELISA sandwich using the Mouse IFN-γ and IL-4 ELISA kits (eBiosciences, San Diego, CA, USA), following the manufacturer’s instructions. All assays were performed in triplicate.

### Protection Experiment

The protection experiments were performed as previously described (16). Briefly, 4 weeks after last vaccination, four mice from each group were challenged by intraperitoneal injection of 10^4^ CFU *B. abortus* 2308 per animal. Two weeks later, infected mice were euthanized and their spleens were homogenized in PBS, with the homogenate serially diluted and cultured in Petri dishes containing agar Columbia supplemented with 5% sheep blood (bioMériex, Santiago, Chile) for 72 h at 37°C. Bacterial counts were recorded and the number of CFU per spleen calculated. This experiment was repeated twice. When the immunizations were initiated, one reference-vaccinated control group was immunized with 1 × 10^8^ CFU *B. abortus* RB51 per mouse. Results are reported as units of protection represented by the difference between mean ± SD of log10 CFU/spleen of the PBS control groups with respect to mean ± SD of log10 CFU/spleen values of experimental groups.

### Statistical Analysis

The immune response in mice was analyzed using a two-way analysis of variance (ANOVA) and the protective response was analyzed using a one-way ANOVA. Data were analyzed using Prism 5.0 (GraphPad software). Differences were considered significant if *P* < 0.05.

## Results

### Epitope Prediction

**Figure 2:**
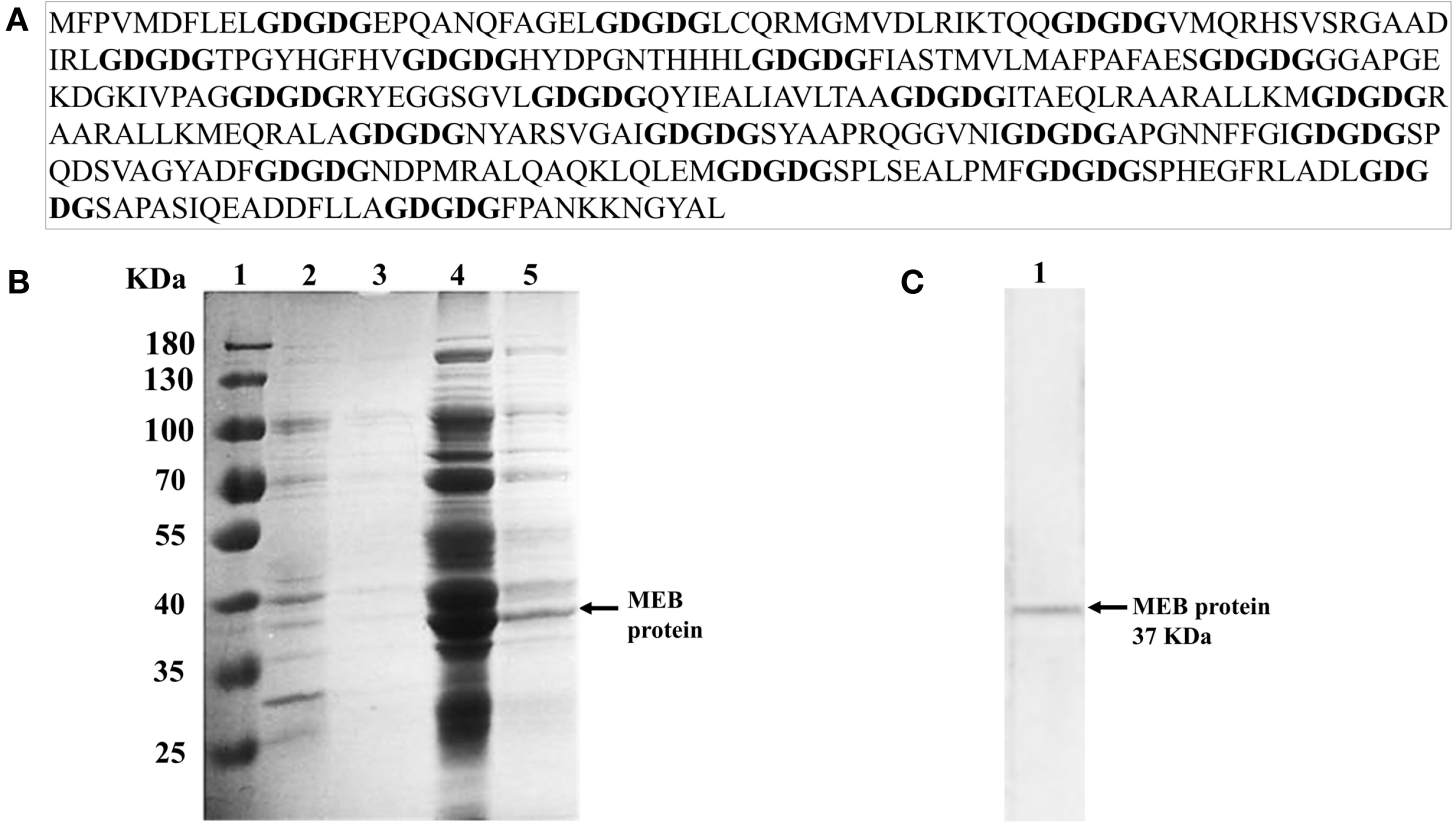
**MEB DNA vaccine design and identification of rMEB protein**. **(A)** Multi-epitope vaccine sequence spaced by GDGDG linker sequence. **(B)** SDS-PAGE analysis of rMEB. Lane 1, marker; lane 2, total proteins obtained from insoluble extract from *Escherichia coli* transformed with pQE80L-MEBe plasmid; lane 3, eluent from Ni^2+^-chelated affinity chromatography of the insoluble extract from *E. coli*; lane 4, total proteins obtained from the soluble extract from *E. coli* transformed with pQE80L-MEBe plasmid; lane 5, eluent from Ni^2+^-chelated affinity chromatography of the soluble extract from *E. coli*. **(C)** Western blot analysis of rMEB with anti-His-tag monoclonal antibody.

Using the Immune Epitope Database (IEDB, www.iedb.org), we identified epitopes suitable for constructing a multi-epitope DNA chimeric vaccine against *B. abortus*. The epitopes identified (Table 1) are specific for MHC class I and MHC class II molecules. However, while all sequences had putative epitopes for MHC class I and class II molecules, the BAB1_0278 ORF only showed epitopes for MHC class I (Table 2). Peptides were selected based on having a lower percentile rank score. Non-redundant peptides were selected to construct the DNA vaccine. An immunodominant peptide of the Cu, Zn superoxide dismutase protein from *B. abortus*, described previously in the literature (39), was also included in the vaccine sequence. Finally, 21 epitopes were used to construct the DNA vaccine. To connect the epitopes, we used a GDGDG linker sequence, a rationally designed sequences used to link multi-epitope vaccines (40). The final design of the multi-epitope vaccine is shown in Figure 2A.

**Table 2:** **Epitope prediction by computer modeling**.

		Allele	Pi	Pf	Peptide	Peptide selected
Cu–Zn SOD	MHC-I	H-2-Ld	60	68	TPGYHGFHV	TPGYHGFHV
		H-2-Kd	92	102	HYDPGNTHHHL	HYDPGNTHHHL
	
	MHC-II	H2-IAd	6	20	IASTMVLMAFPAFAE	FIASTMVLMAFPAFAE
		H2-IAd	5	19	FIASTMVLMAFPAFA
		H2-IAd	7	21	ASTMVLMAFPAFAES

BAB1_0260	MHC-I	H-2-Kd	199	207	NYARSVGAI	NYARSVGAI
		H-2-Kd	311	322	SYAAPRQGGVNI	SYAAPRQGGVNI
		H-2-Ld	114	122	APGNNFFGI	APGNNFFGI
		H-2-Ld	155	166	SPQDSVAGYADF	SPQDSVAGYADF
	
	MHC-II	H2-IAd	353	367	NDPMRALQAQKLQLE	NDPMRALQAQKLQLEM

BAB1_0270	MHC-I	H-2-Ld	35	43	FPVMDFLEL	FPVMDFLEL
		H-2-Ld	128	138	EPQANQFAGEL	EPQANQFAGEL
	
	MHC-II	H2-IAd	46	60	CQRMGMVDLRIKTQQ	LCQRMGMVDLRIKTQQ
		H2-IAd	45	59	LCQRMGMVDLRIKTQ
		H2-IAd	155	169	MQRHSVSRGAADIRL	VMQRHSVSRGAADIRL
		H2-IAd	154	168	VMQRHSVSRGAADIR
		H2-IAd	153	167	DVMQRHSVSRGAADI

BAB1_0273	MHC-I	H-2-Kd	35	43	RYEGGSGVL	RYEGGSGVL
		H-2-Kd	48	60	QYIEALIAVLTAA	QYIEALIAVLTAA
		H-2-Kd	48	56	QYIEALIAV
	
	MHC-II	H2-IAd	3	17	ITAEQLRAARALLKM	ITAEQLRAARALLKM
		H2-IAd	9	23	RAARALLKMEQRALA	RAARALLKMEQRALA

BAB1_0278	MHC-I	H-2-Ld	7	16	SPLSEALPMF	SPLSEALPMF
		H-2-Ld	7	15	SPLSEALPM
		H-2-Ld	21	28	SPHEGFRL	SPHEGFRLADL
		H-2-Ld	21	31	SPHEGFRLADL

BAB1_0278a	MHC-I	H-2-Ld	22	32	FPANKKNGYAL	FPANKKNGYAL
	
	MHC-II	H2-IAd	44	58	SAPASIQEADDFLLA	SAPASIQEADDFLLA

			**Pi**	**Pf**	**Peptide**	**Reference**

Cu–Zn SOD	–	–	75	86	GGAPGEKDGKIVPAG	Tabatabai and Pugh (39)

*Pi, initial position of peptides in each protein; Pf, final position of peptides in each protein*.

### Production of Recombinant Multi-Epitope Protein of *Brucella*

To construct the recombinant protein, *E. coli* BL21 (DE3) cells were transformed with the pQE80L-MEB plasmid and expression of the 6xHis-Tagged protein was induced. The recombinant protein was mainly expressed in the soluble fraction of the transformed bacteria after their sonication (Figure 2B, lane 4). The recombinant protein of *B. abortus* (rMEB) was induced and detected by Western blot. Its weight was ~37 kDa, the expected mass (Figure 2C).

### Humoral Immune Response of Immunized Mice

**Figure 3:**
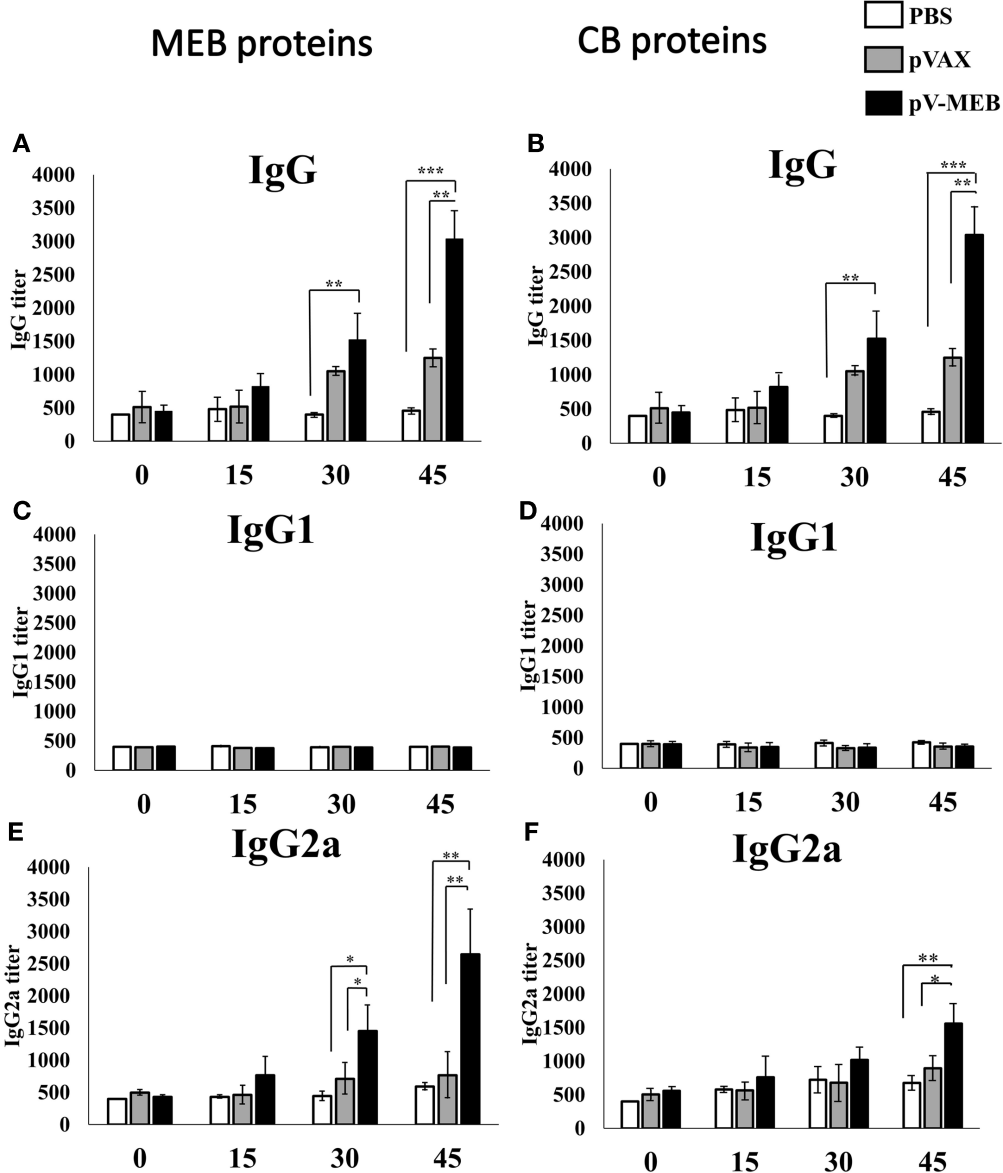
**Titers of specific IgG (A,B), IgG1 (C,D), and immunoglobulin G2a (E,F) production after immunization with recombinant pV-MEB vaccine**. Sera obtained from each group of mice were used for detection of antibodies against purified rMEB proteins **(A,C,D)** and crude *Brucella* proteins **(B,D,F)** by indirect enzyme-linked immunosorbent assay. Sera obtained at days 0, 15, 30, and 45 post-immunization were serially diluted in phosphate buffer saline and used in the assay (*P < 0.05, **P < 0.01, and ***P < 0.001).

Specific antibodies for rMEB and CBPs were measured in order to evaluate the humoral immune response. We performed ELISAs to detect specific IgG, IgG1, and IgG2a antibodies induced in mice against rMEB and CBP. Serum from mice immunized with multi-epitope DNA vaccine for *Brucella* (pV-MEB) contained significant titers of IgG specific for rMEB at 30 days after the first immunization. IgG titers were higher at 45 days, compared to the negative-control groups pVAX and PBS (Figure 3A). The same pattern was observed for IgG against CBPs (Figure 3B). No rMEB- or CBP-specific IgG1 was detected in serum from mice immunized with pV-MEB (Figures 3C,D). Titers of IgG2a antibodies specific for rMEB proteins significantly differed between the pV-MEB groups immunized (*P* < 0.05) only after the second immunization between pV-MEB groups immunized, compared to PBS and pVAX controls (Figure 3E). On the other hand, a significant titer of IgG2a specific for CBP was observed after the third immunization with pV-MEB, compared to PBS and pVAX negative controls (Figure 3F).

### Cellular Immune Response

**Figure 4:**
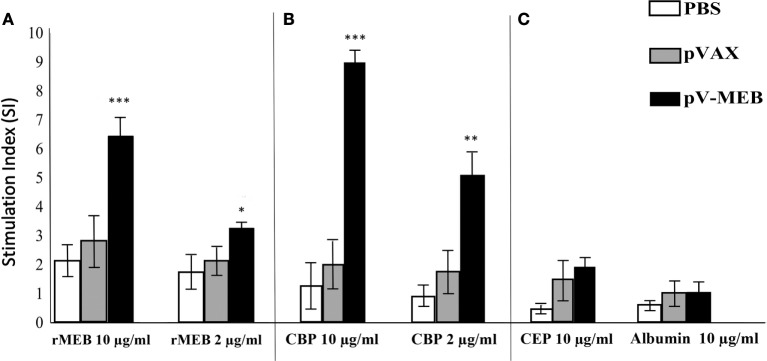
**Lymphocyte proliferation assay after *in vitro* stimulation with (A) 10 or 2 µg/ml recombinant protein, (B) 10 or 2 µg/ml *Brucella abortus* total proteins (CBP), and (C) 10 µg/ml of crude *Escherichia coli* proteins and 10 µg/ml of albumin as control**. Results are shown as mean ± SD of the stimulation index of ^3^H-thymidine, incorporated from mouse splenocytes (*n* = 5) (*P < 0.05, **P < 0.01, and ***P < 0.001).

To evaluate the cellular immune response, splenocytes were obtained from mice immunized with pV-MEB, p-VAX, or PBS at 30 days after the last immunization. *In vitro* stimulation using splenocytes from pV-MEB mice immunized with 10 or 2 µg/ml rMEB protein resulted in a significant increase in cell proliferation in relation to the control group (*P* < 0.001 and *P* < 0.05, respectively; Figure 4A). *In vitro* stimulation using splenocytes from pV-MEB mice immunized with 10 and 2 µg/ml of CBP also induced a significant increase in splenocytes proliferation (*P* < 0.001 and *P* < 0.01, respectively; Figure 4B). In this assay, 10 µg/ml of ConA was used as lymphoproliferation control. It induced a strong lymphoproliferative response in all experimental groups (data not shown). *In vitro* stimulation of splenocytes with 10 µg/ml crude *E. coli* protein and 10 µg/ml albumin did not induce proliferation across the different experimental groups (Figure 4C).

**Figure 5:**
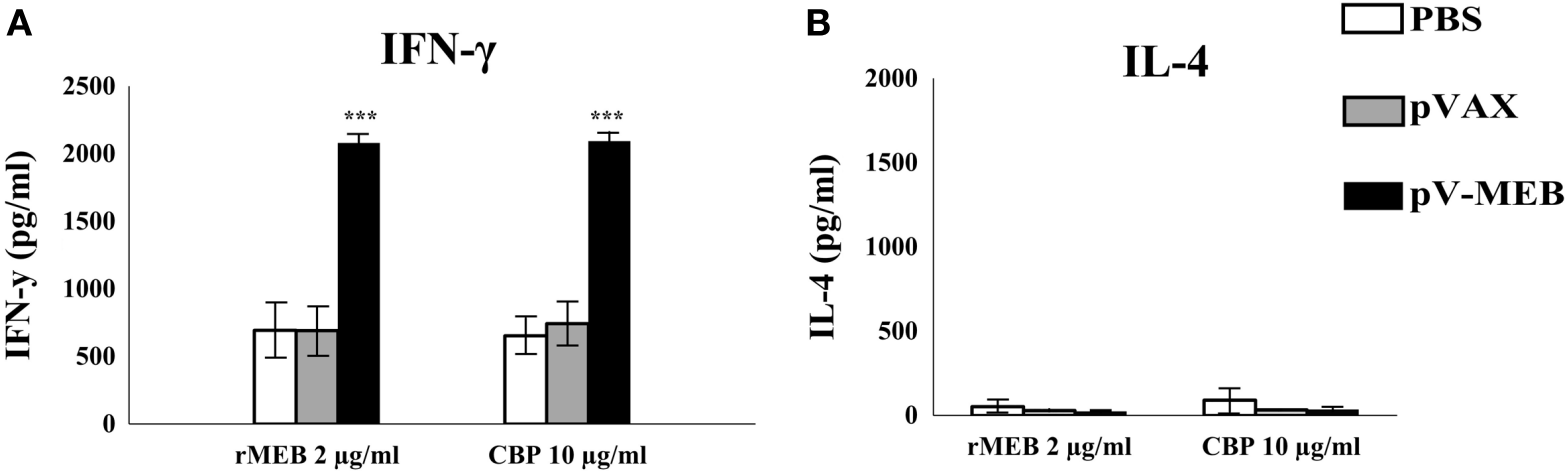
**Determination of IFN-γ (A) or IL-4 (B) production**. Splenocytes from pV-MEB pVAX or phosphate buffer saline (PBS) groups were obtained 30 days after the last immunization, and were re-stimulated *in vitro* with 2 µg/ml of recombinant MEB proteins or 10 µg/ml of crude *Brucella* proteins. Each bar represents the geometric mean ± SD (error bars) of the response in spleen cells from individual mice. ***P < 0.001, statistically significant difference compared to the PBS group.

IFN-γ levels in supernatants from cultures of splenocytes obtained from the pV-MEB immunization group re-stimulated with rMEB or CBP were significantly higher than those in the negative-control groups (*P* < 0.001, respectively) (Figure 5A). There were no significant difference in levels of IL-4 secretion between the experimental and control groups (Figure 5B).

### Protection against Virulent *B. abortus* Challenged

**Figure 6:**
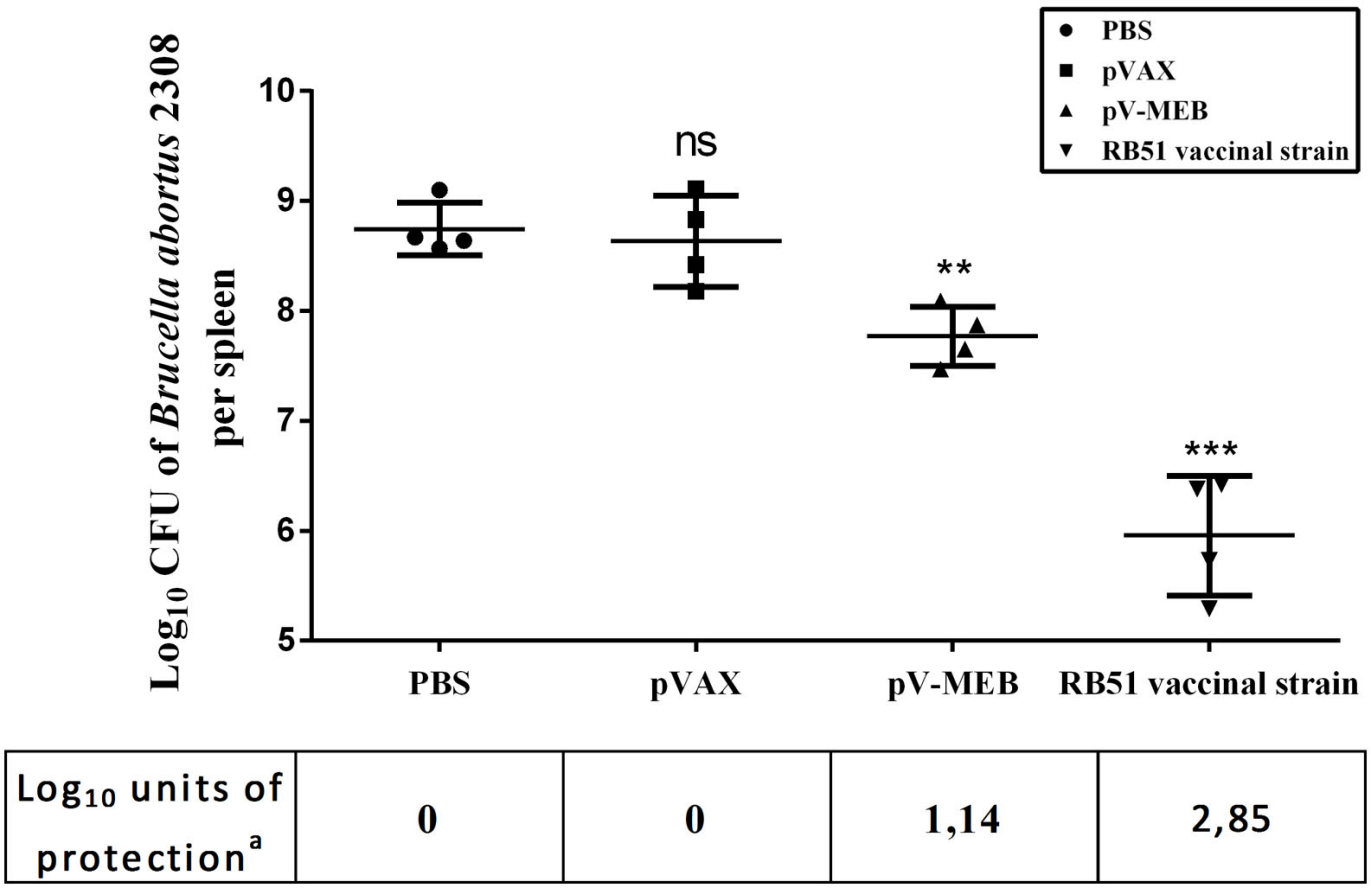
**Protection conferred to BALB/c mice immunized with pV-MEB vaccine against *Brucella abortus* 2308**. Results are shown as mean ± SD of the log_10_ CFU of *B. abortus* 2308 per spleen (*n* = 4), **P < 0.01 and ***P < 0.001. ^a^Units of protection represent the difference between log_10_ CFU values of the phosphate buffer saline group and the log_10_ CFU values of the immunized group.

The protective capacity provided by the pV-MEB DNA vaccine was evaluated 6 weeks after the last immunization. Immunized mice were challenged with 10^4^ CFU *B. abortus* 2308, and after 2 weeks their spleens were removed, homogenized, and cultured. The results showed that pV-MEB DNA vaccine confers protection against *B. abortus* 2308. The DNA vaccine induced 1.14 log_10_ units of protection (*P* > 0.005; Figure 6) compared to the PBS control group. By comparison, vaccination with live *B. abortus* strain RB51 induced 2.85-log units of protection.

## Discussion

Brucellosis is a worldwide zoonotic disease of increasing incidence. Vaccination of livestock is considered the best prevention method, but it is necessary to generate safer and more effective vaccine formulations (41). The availability of bioinformatics tools and databases allow the design of vaccines without the need for *in vitro* manipulation of a pathogenic microorganism. Using “*reverse vaccinology*” approach, *in silico* genomic databases are screened to identify antigenic sequences for new vaccines (42). This allows the identification of antigens that would be difficult using traditional methods (42). It has been observed that recombinant protein vaccines induce a humoral/Th2 immune response and it is suggested that a boost with a protein improve the protective efficacy of the antibodies (43). The effect of DNA vaccines, however, is based toward a Th1 response (44–46).

DNA vaccine have a number of advantages, including ease storage, flexibility of antigen codification, and the presence of CpG motifs, which improve the immune response (45). Mono-antigenic DNA vaccines induce a good immune response, but tend to induce less protection against pathogens compared to poly-antigenic vaccine (47, 48). In the case of *B. abortus*, the three antigens: BCSP31, Cu–Zn SOD, and L7/L12 ribosomal proteins, when giving together as part of a formulation, improve the immune response against pathogenic *B. abortus* (49).

Within the last few years, the use of epitopes in vaccines has become a valid alternative for improving the efficacy of traditional vaccine, based on a “natural” form of the pathogen (50). Multi-epitope peptide DNA vaccines are effective against some viruses (30, 51, 52) and they are potentially effective against some bacteria such as *Helicobacter pylori* (53) and in cancer prevention (54, 55).

Multi-epitope DNA vaccines more faithfully mimic antigen processing and presentation during natural infection (30). In addition, multi-epitope DNA vaccines induce more potent immunoreaction than whole protein vaccine (30). Since the epitopes are derived from multiple antigens and packaged into a relatively small delivery vehicle, the vaccine can induce powerful cross-reactive responses toward multiple antigens and elicit a strong humoral and cellular immune response (56). In this study, we used bioinformatics methods to identify epitopes on antigenic proteins of *B. abortus* and to design a multi-epitope chimeric DNA vaccine. We performed the epitope prediction using bioinformatics resources available online, including NetChop, SYFPEITHI, or BIMAS. However, these database had a 50% prediction assertiveness about the prediction (57). The predictive power of the IEDB has been expanded with the provision of a large number of published epitopes and full-scale MHC-binding peptides (58), so we opted to use the IEDB server. In order to design a rational vaccine against *Brucella*, we focused on finding MHC class I or MHC class II binding peptides known to orchestrate primarily a T-cell immune response, since *B. abortus* is known as a facultative intracellular pathogen (59). We selected 21 dominant epitopes from ORFs present within GI-3 and proteins described as antigens of immunological interest (Table 1). Peptides were selected based on their low percentile score using observed redundant sequences as a further selection criterion, choosing peptides with non-redundant sequences (Table 2). For the theoretical binding of peptides we used the “GDGDG” sequence as spacer (Figure 2A) (40). The introduction of GDGDG spacers does not preclude the possibility that such linear arrangements of epitopes might contain other cryptic epitopes. The presence of this spacer at 15–20 residue intervals might help create some secondary and possibly tertiary structure, thereby facilitating antigen expression (40). Then, we constructed the *B. abortus* multi-epitope chimeric DNA vaccine using of chemical gene synthesis (pV-MEB).

We, next, proceeded to evaluate the immunogenicity and protective efficacy conferred by immunization with the multi-epitope vaccine, peptides present in Cu–Zn superoxide dismutase and the ORFs present within GI-3 (BAB1_0260, BAB1_0270, BAB1_0273, and BAB1_0278) of *B. abortus* (16, 21–26). The results showed that immunization with pV-MEB triggers a MEB-specific humoral and cellular immune response in BALB/c mice. At systemic level pV-MEB promotes the stimulation of MEB-specific IgG2, indicating an adequate induction of a Th1 response. *In vitro* stimulation of splenocytes from pV-MEB immunized mice induced the highest proliferation in response to antigen, confirming the *in vivo* translation of the *MEB* synthetic gene and subsequent induction of a cell-mediated immune response. We used albumin and *E. coli* proteins as control of proliferation and in both cases splenocytes did not proliferate. Therefore, the immune response inducing by pV-MEB DNA vaccine was specific to MEB protein and *Brucella* antigens. Antigen-stimulated splenocytes from vaccinated mice produced IFN-γ. The level of IFN-γ and the *in vitro* proliferation of splenocytes stimulated by MEB recombinant protein demonstrated that pV-MEB DNA vaccine induces a strong immunoreaction and a polarized Th1 response against *Brucella* infection, which is associated to effective clearance of intracellular pathogens, so essential feature for a *Brucella* vaccine (7, 59). The immunogenicity induced by pV-MEB DNA recombinant plasmid was evaluated by challenging immunized mice with *B. abortus* 2308 strain. Our results confirmed that immunization with pV-MEB induced immunogenicity associated with significant protection, but protection induced by attenuated *B. abortus* RB51 was more robust.

In conclusion, we have shown that a *B. abortus* multi-epitope chimeric DNA vaccine (pV-MEB) elicits strong humoral and cellular protective immunity. Future studies must include an array of epitopes or combination of peptides and adjuvants as alternatives to conventional vaccine design. This study provides a starting point for the development of multi-epitope DNA vaccines against *B. abortus*.

## Author Contributions

EE: construction of recombinant plasmid, analysis *in silico* of determination of epitopes, evaluation of immune response, evaluation of antibodies specificity by ELISA, and writing and discussion of the results. DS: immunization trail, lymphocyte proliferation assays, and protection experiment. AO: programming and monitoring the experiments, performing the analysis, and discussion and writing the manuscript. AO is principal investigator at the FONDECYT grant that funded this work.

## Conflict of Interest Statement

The authors declare that the research was conducted in the absence of any commercial or financial relationships that could be construed as a potential conflict of interest.
